# Pathology

**Published:** 1890-11-15

**Authors:** 


### THE LONDON POST-GRADUATE COURSE AT THE PADDINGTON INFIRMARY.

### PATHOLOGY.

Dr. Savill in commencing his demonstration spoke of the results of inflammation. These, he said, may be divided into five groups: 1, resolution; 2, organisation, which may be of a higher or lower type of tissue; 3, degeneration, which may be granular or fatty (suppuration); 4, necrosis (or gangrene), which occurs if the process is extensive or very intense; 5, metastasis, a term used by the old authors and instanced in the inflammation of the testes which occurs in mumps, or in some cases of acute rheumatism, when one joint gets well and another becomes affected. Many of these metastatic phenomena are now explained as bacteriological. In any given case which of the five results of inflammation occurs depends upon three factors: 1, the tissue or organ involved; 2, the age of the patient; and 3, the state of the person's health at the time being. After this preliminary discussion a few words were said in conclusion on the subject of chronic Bright's disease, which was described in the previous demonstration; namely, the histology of the disease. First, taking the smooth white kidney, in which the main part of the changes are in the epithelium of the tubules. It is frequently a sequela of acute nephritis, in which case the epithelium is very swollen and granular, and also increased in quantity, the tubules become filled with epithelium and epithelial débris. In the post-scarlatinal form of this disease changes in the vessels are also described, consisting in a thickening both of the vessels and also of the glomeruli, and hence called a glomerulo-nephritis; in these cases the epithelial cells sometimes contain lime salts. In this large white kidney the stroma, as a rule, is not much changed; in the later stages, however, there may be some increase, which, again, still later leads to some contraction of the whole organ. Secondly, the gouty kidney; here the epithelium which happens to remain is very granular, but most of it drops out in mounting for the microscope; the main change being in the stroma, which becomes very much increased, and in the later stages of the disease the whole gland is much contracted. The vessels and Malphigian bodies are much thickened; the same also occurs with the basement membrane of the glomeruli. Thirdly, the waxy kidney. In this form the parenchyma is sometimes slightly granular, but is rarely involved in the amyloid disease, which affects the vessels, especially those of the Malphigian tufts. These in an early stage of the disease appear as glistening points, to be seen with the naked eye. In the later stages the basement membrane of the tubules becomes affected, and the stroma may be increased. With regard to the nature of the waxy or lardaceous material, this was originally described by Virchow as an amyloid material from the belief that it was of a starchy nature on account of its blue reaction with iodine. Since then, it has been proved not to be of an amyloid, but of a proteid nature, and contains about 15 per cent. of nitrogen. So, also, it has several reactions the same as those for proteids, such as the colour reaction with acids and alkalies; also it is soluble in dilute alkalies, and is reprecipitated by dilute acids. Thus it is of an albuminoid rather than of an amyloid nature. The best test for this body in the post-mortem room is the iodine solution with a small quantity of iodide of potassium dissolved in it. With this solution the amyloid substance stains of a deep mahogany-brown colour. It is very difficult, and often quite impossible, to draw a sharp and fast distinction between all of these three forms of kidney disease, each variety depending upon the tissue which is primarily affected, that is to say, the epithelium, the stroma, or the vessels. The question also as to whether the large white kidney and the gouty kidney are but different stages in one and the same disease is a very hard one to answer. The German authors generally regard them as two stages of the same disease, but there are some points which suggest that we have here two distinct diseases, and this is the position taken by most of the English writers. From the pathological point there are a few marked differences—in the first place, the tendency of the epithelium of the large white kidney to become fatty, which is not the case with the gouty kidney, also the stroma in the latter form is very much increased; on the other hand, although the large white kidney may in some case be somewhat decreased in size, yet there is never that marked disproportion between the two tissues, that is, the parenchyma and stroma, as in the gouty kidney. Clinically there are also some points of distinction. With the large white kidney there is a marked tendency to dropsy, which is not the case in the gouty one. Dr. Johnson collected 33 fatal cases of granular kidney, and in 19 there was neither dropsy nor any history of previous dropsy. The ætiology also gives some distinctions, the large white being frequently a sequela of acute parenchymatous nephritis, often, for instance, following scarlet fever, diphtheria, or erysipelas, or, in fact, almost any fever; or as the consequences of a chill, especially in elderly people; so also an injury, spreading from some bladder mischief, or some drug, such as cantharides or turpentine. The text-books give also mercury, but there is not much to support this theory. The disease may also come on irrespective of an acute attack. The causes may be summed up under the following headings: Alcohol, cardiac disease, pregnancy, especially if to any of these is super-added a chill. With regard to the causes of the gouty kidney, the most important is gout, using the term in its widest significance, including, for instance, the gouty diathesis, that is, persons with a sluggish digestion, lithates in the urine, and in some cases urates of soda in the epithelium of the tubules of the kidney. Age is also a condition, thus it is especially frequent between forty and fifty years; and, thirdly, occupation; particularly is it common in those with none, and who lead sedentary lives; so also workers in lead, that is, painters, &c. In the last place, the lardaceous kidney is produced, along with similar changes in other organs and parts of the body (1) after prolonged suppuration, especially phthisis, dysentery, ozoena; (2) diseases of the bones, with or without suppuration; (3) constitutional syphilis; and (4) ague. The question of the relation of the gouty kidney to the cardio-vascular changes is ever a very difficult one to answer. In the first place, with regard to the heart itself, Bright recognised that in many cases of kidney disease there was some hypertrophy of the left ventricle. The left ventricle, in fact, is larger than normal in ninety per cent. of the cases examined, and this is an hypertrophy without any valvular disease accompanying it. The explanation given by Bright, and accepted by many other writers, is that this change is due to some poison in the blood, which acts upon and causes hypertrophy of the muscular fibres; but if this were the explanation, it is very difficult to see why the other muscles in the body should not atrophy in this complaint. There is frequently a widespread change, consisting in the thickening of the coats of the vessels throughout the body; but this is not found in all cases, nor necessarily in those with most marked kidney mischief. Johnson described the change as a thickening of the muscular coat. Later, Gull and Sutton said the increase was in the tunica adventitia, and was of a fibro-hyaline nature; but there is reason to believe that the change occurs in both the media and also in the adventitia, at least it appears to be so when the sections are stained for a long time, and so the nuclei of the fibrous tissues are brought out. Finally, a case described was that of a woman, M. W., 71 years of age, with a history, that for the last two and a half years she had suffered from cough, and had twice been in the infirmary, the first time in January of this year, after which she left improved. She never got quite well, and returned to the infirmary again in October. The symptoms were those of chronic bronchitis, with a dilated right heart, and on admission, besides the diffuse bronchitic râles, there were dulness with tubular breathing and bronchophony about the angle of the left scapula. The temperature was 100·6, and for the rest of her life it was normal or sub-normal. She died on November 2nd. At the *post-mortem*, the right side of the heart was much dilated, and the tricuspid orifice admitted four fingers and the thumb; there was but slight roughness of the valves. The left lung weighed 12 ounces, there was some consolidation at the base, and this area was darker than natural; there was an absence of the usual crepitant feel, the substance was firm and hard, and there was some emphysema at the margins of both lungs, together with considerable congestion of the lining membrane of the bronchi, the consolidated patch showing all the signs of chronic interstitial pneumonia. The right lung weighed 15 ounces, and presented no obvious lesion. Under the microscope there was seen to be a considerable amount of connective tissue in the region of consolidation of the left lung, not only in the interlobular spaces, but also between the air cells, and the disease had apparently started in the region of the small vessels. The kidneys were congested, especially the glomeruli, their substance somewhat firm, each weighing 4 ounces. The liver weighed 42½ ounces; it was congested, and its substance very friable and more or less mottled and fatty. The spleen was apparently healthy.

---

Sir Edward Watkin complains somewhat sorrowfully that the South Eastern Railway Company has only received abuse for its efforts to enable working men to go into the country. This puts railway companies before us in a new and strange light; we have grievously wronged them by not supposing them to be particularly sensitive bodies.

